# 3T contrast-enhanced whole heart coronary MRA using 32-channel cardiac coils for the detection of coronary artery disease

**DOI:** 10.1186/1532-429X-11-S1-O5

**Published:** 2009-01-28

**Authors:** Qi Yang, Kuncheng Li, Xiaoming Bi, Jing An, Renate Jerecic, Debiao Li

**Affiliations:** 1 Xuanwu hospital, Beijing, PR China; 2 Siemens Medical Solutions, Chicago, IL USA; 3 Siemens Mindit Magnetic Resonance Ltd, Shenzhen, PR China; 4 Department of Radiology, Northwestern University, Chicago, IL USA

**Keywords:** Acceleration Factor, Suspected Coronary Artery Disease, Image Quality Score, Coronary Artery Stenos, Significant Stenos

## Introduction

In recent years, improved gradient performance and radiofrequency (RF) receiving coils and advanced data acquisition techniques including navigator gating and parallel imaging allowed non-invasive whole-heart coronary imaging. Previous studies have shown that 3.0 T is a promising platform for the detection of significant coronary artery stenoses with contrast-enhanced data acquisition. However, the imaging time (~10 minutes) and spatial resolution (1.3 × 1.3 × 1.3 mm^3^) remain major limitations [1]. Newly developed 32-channel cardiac coils allow greater acceleration factors and thus reduced imaging time and higher spatial resolution [2].

## Purpose

To evaluate the feasibility and diagnostic accuracy of 3 T contrast-enhanced whole-heart coronary MRA using 32-channel cardiac coils. The imaging time, image quality score, and diagnostic accuracy were evaluated in consecutive patients with suspected coronary artery disease.

## Methods

20 patients with suspected coronary artery disease who were scheduled for x-ray coronary angiography (mean age 68 ± 14 years, 11 males) underwent MRA at 3 T (MAGNETOM Tim Trio, Siemens) after informed consent was obtained. Contrast-enhanced coronary MRA was also performed in 5 patients who were scheduled for 64-slice coronary CTA. The imaging technique was an ECG-triggered, navigator-gated, inversion-recovery, segmented gradient-echo sequence. A 32-channel matrix coil was used for data acquisition. To reduce imaging time, parallel acquisition (GRAPPA) was used in the phase-encoding direction with an acceleration factor of three. Imaging parameters included: voxel size 0.55 × 0.55 × 0.65 mm^3^ (interpolated from 1.1 × 1.1 × 1.3 mm^3^), TR/TE = 3.3/1.5 msec, flip angle = 20°, bandwidth = 700 Hz/pixel. Contrast agent (0.15 mmol/kg body weight, Multihance, Bracco Imaging SpA, Italy) was intravenously administered at a rate of 0.3 ml/sec. The diagnostic accuracy of MRA in detecting significant stenoses (≥ 50%) with the intention to diagnose method was evaluated on a per-segment basis using x-ray angiography as the reference, non-assessable segments were considered to be false-negative or false-positive, respectively.

## Results

**Figure 1:**
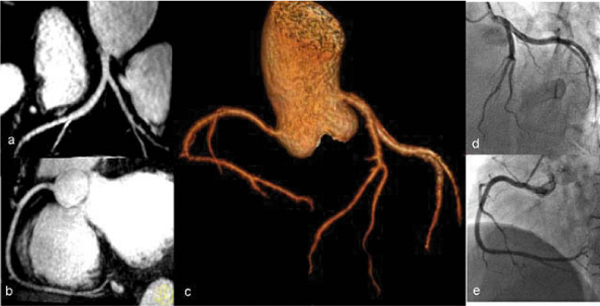
**3 T Coronary MR image of a 50-year-old patient**. Reformatted images (**a**, **b**) and volume rendering image demonstrates normal RCA, LM, LAD. Better visualization of the entire coronary artery tree after removing the background of myocardium, long segments of all major coronary arteries are well depicted and correlate well with X-ray angiography (**d**, **e**).

Whole-heart coronary MRA was successfully completed in 19 of 20 (95%) patients who were scheduled for x-ray coronary angiography and in 5 patients who were scheduled for 64-slice coronary CTA. The averaged imaging time with 32-channel cardiac coils was 6.2 ± 1.3 min. The sensitivity, specificity, positive predictive value, and negative predictive value of coronary MRA for detecting significant stenoses were 81% (62–94%), 96% (92–98%), 71% (52–86%), 98% (94–99%), respectively, on a per-segment basis. Figure 1.

## Conclusion

Combined with dedicated 32-channel cardiac coils, parallel imaging with higher acceleration factors allows improvements in imaging speed, study success rate, and reduced dose of the contrast agent when compared with conventional 12-channel coils. Higher study success rate achieved by 32-channel coils substantially improved overall accuracy of coronary MRA in detecting coronary artery disease when using the intention to diagnose method.
